# Non-Native Non-*Apis* Bees Are More Abundant on Non-Native Versus Native Flowering Woody Landscape Plants

**DOI:** 10.3390/insects13030238

**Published:** 2022-02-28

**Authors:** Daniel A. Potter, Bernadette M. Mach

**Affiliations:** Department of Entomology, University of Kentucky, Lexington, KY 40546-0091, USA; b.mach@ufl.edu

**Keywords:** Apoidea, pollinator conservation, urban landscape, non-native plant, *Megachile sculpturalis*, *Osmia*, invasive species

## Abstract

**Simple Summary:**

Bees and other pollinators play a vital role in food production and natural ecosystems. Native bee populations are declining due in part to habitat loss. Individuals can help bees by landscaping with plants that provide pollen and nectar. Most information on bee-friendly plants concerns herbaceous ornamentals, but flowering trees and shrubs, too, can provide food for urban bees. Conservation organizations recommend landscaping mainly with native plants to support native bees, but some studies suggest that including some non-invasive non-native plants that bloom earlier or later than native plants can help support bees when resources from native plants are scarce. That strategy might backfire, however, if such plants disproportionately host invasive bee species. This study tested that hypothesis by identifying all non-native bees among 11,275 bees previously collected from 45 species of flowering woody plants across hundreds of urban sites. Besides the ubiquitous honey bee, six other non-native bee species comprised 2.9% of the total collection. Two alien species considered to have invasive tendencies by outcompeting native bees were more abundant on non-native plants. Planting their favored hosts might facilitate those bees’ spread in urban areas. Pros and cons of non-native woody landscape plants for urban bee conservation warrant further study.

**Abstract:**

Urban ecosystems can support diverse communities of wild native bees. Because bloom times are conserved by geographic origin, incorporating some non-invasive non-native plants in urban landscapes can extend the flowering season and help support bees and other pollinators during periods when floral resources from native plants are limiting. A caveat, though, is the possibility that non-native plants might disproportionately host non-native, potentially invasive bee species. We tested that hypothesis by identifying all non-native bees among 11,275 total bees previously collected from 45 species of flowering woody landscape plants across 213 urban sites. Honey bees, *Apis mellifera* L., accounted for 22% of the total bees and 88.6% of the non-native bees in the collections. Six other non-native bee species, accounting for 2.86% of the total, were found on 16 non-native and 11 native woody plant species. Non-*Apis* non-native bees in total, and *Osmia taurus* Smith and *Megachile sculpturalis* (Smith), the two most abundant species, were significantly more abundant on non-native versus native plants. Planting of favored non-native hosts could potentially facilitate establishment and spread of non-*Apis* non-native bees in urban areas. Our host records may be useful for tracking those bees’ distribution in their introduced geographical ranges.

## 1. Introduction

High prevalence of non-native species is a defining feature of urban floras [1,2]. Non-native plant species and cultivars dominate the horticulture market [3] and are popular for reasons that include growth form, foliar and floral characteristics, resistance to insect pests and diseases, and tolerance of abiotic stressors associated with urbanization [2]. For sustainable landscapes, the benefits of such plants should be weighed against their potential detriment to ecosystem function [4]. For example, urban landscapes dominated by non–native plants (i.e., those that evolved outside of local food webs) may be inhospitable to native caterpillars and thus to birds that rear their young on insect prey [5]. In contrast, many species of cultivated non-native plants provide floral resources to urban bees [6,7,8,9,10,11].

Seasonal timing of bloom tends to be conserved by biogeographic origin, with plants introduced into new regions retaining the phenology of their natal provenance [12,13]. Thus, some authors, e.g., [9,10,14,15], suggest that, in anthropogenically-transformed managed urban gardens and landscapes, incorporating some non-invasive non-native plant species can help to support pollinators by ensuring succession of overlapping bloom periods, supporting seasonal specialists, and providing floral resources before and after peak bloom of native plants. Most urban bee species are polylectic and readily incorporate pollen and nectar from novel plants into their diets [13,16,17]. One potential caveat, though, is that some non-native bees preferentially forage on plants of their own provenance [18,19,20,21].

At least 55 non-native bee species have been purposefully or accidentally introduced to North America [22]. While such bees provide pollination services [23], they may also spread pathogens to native bees or compete with them for food or nesting sites [18,22,24,25]. Those potential effects are concerning because there is growing evidence of decline in some wild bee populations [26,27,28]. In urban ecosystems, local abundance of the honey bee (*A. mellifera*; Apidae), a dietary generalist, is more likely affected by intensity of urban apiculture [24], but if other non-native bees prefer foraging on plants of their own provenance, planting such species could potentially facilitate those bees’ establishment in urban areas [21,24,29,30]. In a worst-case scenario, preference of non-native bees for non-native plants could promote invasive mutualisms whereby each facilitates the other’s spread [30,31,32].

Previously [9] we sampled bees from the blooms of 45 species of flowering woody landscape plants (trees and shrubs; Supplemental Table S1) across 213 established urban and suburban landscape sites in Kentucky and southern Ohio, USA. In total, 11,275 bees were collected, pinned, and identified to genus. We found significant plant species effects and variation in seasonal activity of particular bee families and genera, but no differences in overall bee visitation or bee genus diversity between native and non-native plants [9]. Although that study identified many bee-attractive, non-invasive non-native woody landscape plants, without species-level resolution it was not possible to determine if those plants disproportionately host non-native non-*Apis* bees. For the present analysis, we searched for and identified all non-native bees in those collections to determine species present, and their relative abundance and host associations, in order to test the hypothesis that non-native bees are more abundant on non-native than on native woody landscape plants.

## 2. Materials and Methods

### 2.1. Background on Sample Sites

Sites sampled by Mach and Potter [9] included residential and commercial landscapes, street verges, medians and parking lots, cemeteries, campuses, and urban arboreta. Some sites contained a single tree or shrub of the focal species; others contained a grouping of that species. Same-species sites were separated by >1 km to limit overlap of bee populations. We sampled from five sites for each of 35 plant species, four sites each for 8 plant species, and three sites for two harder-to-find species. At each site, sampling involved netting or hand-collecting the first 50 bees observed foraging on the focal plant species during its peak bloom. The 45 species of trees and shrubs varied in stature, seasonal timing of flowering, and number and size of blooms. Some yielded a 50-bee sample in just a few minutes, whereas others required longer. Therefore, the analysis herein is based on the number of non-native bees in standardized sample sizes, not the relative attractiveness of the different plant species.

### 2.2. Bee Identification

We started by compiling a list of non-native bee species known to occur in the eastern United States [22,33], eliminating dietary specialists not associated with any of the woody plants in the survey. Initial bee identifications were made using the Discover Life keys [34] which include high-quality reference images along with reprinted taxonomic descriptions from the primary literature. We also consulted the primary literature to confirm key identifying features and determine if there were other helpful notes on identification, distribution, flight timing, or floral hosts of those non-native bees. All of the non-native bees potentially present in our region, and on plant species we sampled, are readily distinguishable by key features including hair color, body texture (e.g., presence/absence of pits), clypeal and mandible structure, size, and flight timing. We then searched the collections for specimens of non-native bee species known to occur in or having the potential to spread to Kentucky from other eastern states, sorting through all specimens in the appropriate genus; e.g., all *Andrena* if searching for *Andrena wilkella* (Kirby). Some genera could be sorted by size (e.g., *A. wilkella* body length is 9–12 mm, so much smaller specimens could be eliminated) or by characteristic features (e.g., horns on *Osmia cornifrons* (Radoszkowski) and *Osmia taurus*. Identification was then verified by consulting the original published keys and taxonomic literature [35,36,37,38,39]. Voucher specimens are deposited in the University of Kentucky’s Department of Entomology Insect Collection.

### 2.3. Statistical Analysis

Abundance of particular non-native bee taxa was analyzed for main effects of plant species, plant family (as a proxy for plant species due to limited degrees of freedom), provenance (native or non-native), and plant type (tree or shrub) using the general linear models procedure (SAS, Version 9.4; SAS Institute, Cary, NC, USA), with mean separation by least square means. Plants were coded as non-native if they had mixed origins. An overall analysis including all non-native bees except *A. mellifera* was conducted for each main effect. Individual species (*A. mellifera*, *Megachile sculpturalis* Smith, and *O. taurus*) were further analyzed for each main effect. Four other non-native bee species (*A. wilkella*, *Hylaeus punctatus* (Brullé), *Megachile rotundata* (F.) and *O. cornifrons*) were not analyzed separately due to small sample size.

## 3. Results

We found and identified 2834 individuals of non-native bee species which accounted for 25.1% of the 11,275 bees previously sampled from flowering woody landscape plants. *Apis mellifera* was the most abundant non-native bee species; 2512 individuals collected from 44 plant species accounted for 22% of the total bees sampled, and 88.6% of the non-native bees (Table 1). Besides *A*. *mellifera*, 322 individuals of other non-native bee species were found on 27 different plant species, accounting for 2.86% of the total bees sampled (Table 1). Non-*Apis* non-native bees belonged to three families: Megachilidae (four species), Andrenidae (one species), and Colletidae (one species). The most abundant non-*Apis* non-native bee was *O. taurus* (*n* = 136 individuals), followed by *M. sculpturalis* (*n* = 97), *M. rotundata* (*n* = 37), *O. cornifrons* (*n* = 28), *A. wilkella* (*n* = 25), and *H. punctatus* (*n* = 2). When more than one specimen of a particular bee species was collected from a particular host, in most cases that bee was found on that plant species at multiple sites (Supplemental Table S2). Considering the subset of 5789 individual bees in the samples from non-native plants (Table 1), most (73.7%) were of native bee species; the remainder consisted of *Apis mellifera* (21.5%) and non-A*pis* non-native bees (4.8%).

**Figure 1:**
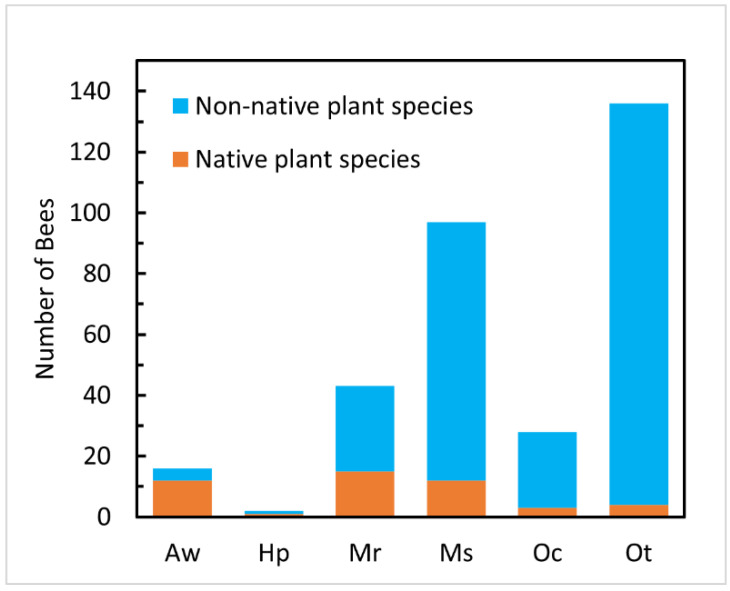
Total number of individuals of six non-*Apis* non-native bee species in ca. 50-bee samples from flowering woody landscape plants, showing proportion of each bee species collected on non-native (*n* = 24) or native (*n* = 21) plant species. Aw, *Andrena wilkella*; Hp, *Hylaeus punctatus*; Mr, *Megachile rotundata*; Ms, *Megachile sculpturalis*; Oc, *Osmia cornifrons*; Ot, *Osmia taurus*. Those bees together accounted for 2.86% (322/11,275) of all bees sampled.

Plant provenance was a significant factor for abundance of all non-*Apis* non-native bees analyzed (Table 2). Overall, non-*Apis* non-native bees were more abundant on non-native woody landscape plants than on native ones, with 275 individuals sampled from 17 (out of 24) species of non-native plants and only 47 individuals sampled from 10 (out of 21) species of native plants (Figure 1). Plant species and plant family were significant factors in the overall abundance of non-native bees and were the only significant factors for abundance of *A*. *mellifera* (Table 2). Plant type (tree or shrub) was non-significant except for *O. taurus* which was more abundant in samples from trees than shrubs (98 versus 38 total individuals, respectively; Table 2).

*Apis mellifera*, a dietary generalist [40], was equally abundant on natives and non-natives, with 1273 individuals sampled from 21 native plant species and 1239 individuals sampled from 23 non-native plants (Table 1). It was absent only in the samples from *Cercis canadensis* L., an early-blooming native tree.

*Andrena wilkella*, native to Europe and northern Asia, is the only non-native andrenid known to be established in the United States [22]. We found it on seven native and five non-native plants whose bloom times ranged from May to late summer (Table 1). *Hylaeus punctatus*, a European species, was the only non-native colletid in our samples. Two specimens were collected, one each from *Aesculus parviflora* Walter (native) and *Koelreuteria paniculata* Laxm. (non-native) (Table 1).

Nineteen of the 40 non-native bee species known to have been introduced into the continental United States belong to the solitary, cavity-nesting family Megachilidae [19]. Four megachilids, *O. taurus*, *O. cornifrons*, *M. rotundata*, and *M. sculpturalis* accounted for about 92% of the non-*Apis* non–native bees we collected (Table 1). *Osmia taurus*, native to eastern Asia, was collected from 10 species of early-season blooming plants, nine of them Asian or hybrids of Asian and North American species (Table 1). Phenology and host associations of *O. cornifrons*, a species intentionally introduced from Japan in the 1970s for crop pollination, are similar to those of *O. taurus* (Table 1). We found *M. rotundata* on seven plant species, with 68% of the specimens from late-blooming non-native plants (Table 1). *Megachile sculpturalis* is a large species native to eastern Asia [20]. We collected it on six of the 45 woody plant species sampled, including 12 specimens collected from two native trees, *A. parviflora* and *Oxydendron arboreum* (L.), and 85 specimens from *K. paniculata*, *Tetradium daniellii* Lour., *Vitex agnus–castus* L., and *Maackia amurensis* Rupr. and Maxim.. The latter four species, all of Asian origin and blooming mid- to late-summer, accounted for 88% of the *M. sculpturalis* collected.

## 4. Discussion

Establishment and spread of non-native bees can adversely affect native bee communities through competition for floral resources or nesting sites, co–invasion with shared pathogens, introgressive hybridization, or disruption of pollination networks [18,23,25,41]. Most purposeful introductions have involved managed eusocial bees (*Apis*, *Bombus* spp.) used for their agricultural pollination services, and the literature is often inconclusive or mixed about the effects of such introductions [22,42]. Much less is known about how solitary, non-managed non-native bees affect native bees and associated ecosystems. Indeed, only eight of the 67 documented species of non-*Apis* or *Bombus* spp. non-native bees worldwide have associated empirical studies that tested for such effects [22]. Nonetheless, some recent studies found negative correlations between bowl-trap catch abundance of native and non–native non-*Apis* bees in particular locales [24,43].

The present analysis supports the hypothesis that, compared to native woody landscape plants, non-native plants hosted higher numbers of non-native bees. Similar patterns have been documented for non-native bees foraging on herbaceous plants; e.g., European bumble bees introduced into New Zealand showed strong foraging preference for European-origin plants [44]. Similarly, native bee species were common on both native and non-native herbaceous plants in California urban gardens, but substantially more non-native bee species visited non-native plants than native ones [10]. All of the non-native woody plants we sampled are also visited by native bees [9]. Moreover, across all 45 woody plant species, 80% (12/15) of the ones that bloomed before 1 April or after 1 July are non-native, supporting the view that non-native garden and woody landscape plants can provide food for urban bees during periods when floral resources from native plants may be limiting [9,14,15,22].

None of the non-native cultivated plants we sampled from are considered invasive or potentially harmful in the United States [45]. However, such plants might serve as stepping stones for the spread of potentially invasive non-native bees? Considering the non-native bees in our samples, *A. wilkella*, which was probably accidentally introduced from Europe in ship ballast, is already common and widespread throughout the north-central and northeastern United States and southern Canada [35]. *Hylaeus punctatus* is expanding its range from several points of introduction and has potential to spread throughout North America [38]. There are no published reports of negative ecological impacts from either of those species [22]. *Megachile rotundata*, detected in the United States by the early 1940s, is now managed and widely used to pollinate alfalfa and other field crops. It reportedly favors agricultural or other disturbed sites so is unlikely to pose a competitive threat to native pollinators [46], a conclusion consistent with assessments from elsewhere in the bees’ introduced range [47,48]. All three of the aforementioned species are polylectic [31], foraging on both native and non-native plants, so incorporating non-native flowering woody plants into urban landscapes is unlikely to facilitate those bees’ spread.

Two Asian mason bees, *O. cornifrons* and *O. taurus*, have recently established in the eastern United States; the former was intentionally introduced in the 1970s for fruit crop pollination, and the latter, apparently an accidental introduction, was first documented in 2002 [43]. Bowl-trap records for the two non-native and six native *Osmia* species in the Mid-Atlantic Region from 2003 to 2017 revealed that *O. cornifrons* catch abundance was stable, but *O. taurus* increased by 800%, with concurrent declines for all six native congeners [43]. Reasons for those changes are unclear, but there is overlap in the bees’ floral preferences, and *O. taurus* and *O. cornifrons* both compete with native *Osmia* spp. and other cavity-nesting native bees for nesting sites [49]. Feral populations of *O. cornifrons* in the United States host fungal pathogens originally reported from Japan [50], but whether that pathogen has been introduced into native *Osmia* populations has not been established. Climate or habitat changes may also be favoring the introduced species [43]. *Osmia cornifrons*, a mesolectic species, favors pollen from rosaceous plants from its native East-Asian provenance, but also broadly uses pollen from European and North American species of *Prunus*, *Rubus*, and *Cercis* [21]. Floral preferences of *O. taurus* have yet to be documented in North America [43]. We collected it on many of the same spring-blooming plants that hosted *O. cornifrons*; e.g., *Prunus¸*and *Malus* spp., and native *Cercis canadensis* (eastern redbud. Those hosts are already widespread in urban landscapes. *Viburnum* × *burkwoodii* was especially attractive to *O. taurus*, so if it becomes more widely planted, it could possibly facilitate that bee’s adaptation to urban settings.

*Megachile sculpturalis* probably poses the greatest immediate invasion threat of the non-native bee species we collected. Native to the Eastern Palearctic (China, Japan, Korea, Taiwan), it was first reported outside its native range in 1994, in North Carolina USA [29], it rapidly spread throughout the eastern United States and southeastern Canada and is established at least as far west as Texas and Kansas [51,52]. It was first found in Europe in 2008 where it is rapidly expanding its range [20,53,54]. It is cavity-nesting, competes with native bees for nesting sites [54], and has been observed forcibly evicting native *Xylocopa* and *Osmia* spp. and occupying their nests [20,55]. Previously-reported floral host associations of *M. sculpturalis* consist of at least 43 species of woody and herbaceous plants in 21 families [52], including four of the six woody landscape plants (*K. paniculata*, *O*. *arboretum*, *T. daniellii*, and *V. agnus-castus*) reported herein. Despite being polylectic for nectar, it shows preference for pollen from plants from its own East-Asian origin [20,54]. We collected it on *M. amurensis* (Amur maackia), *T. daniellii* (bee bee tree), and *K. paniculata* (goldenrain tree), all native to East Asia, on *Vitex agnus–castus* (chaste tree) which is native to the arid and semi-arid Mediterranean and Western Asia, and on one native North American tree, *O. arboretum* (sourwood). Planting of its favored non-native hosts as ornamental trees could help *M. sculpturalis* to establish populations in urban areas and facilitate its continued range expansion in North America [29,51,52], Europe [20,53,54], and elsewhere. Moreover, because of its inordinately large size and distinctive coloration, unlike most other species of Anthophila it can be identified without specialized taxonomic skills [53,54]. Thus, its association with certain Asian-origin woody landscape plants may help to focus plant-site observations by citizen-scientists participating in studies to track the bee’s spread, e.g., [20,53,54]. The other non-native bees would be difficult for a non-specialist to identify, but documenting their associations with particular woody landscape plants may be useful to scientists concerned with sampling and tracking those bees’ distribution within their introduced geographical ranges.

Both *M*. *sculpturalis* and *O*. *taurus* were statistically more abundant on non-native woody plants of predominantly Asian origin, and *O*. *cornifrons*, too, was strongly associated with Asian plants. It is unknown to what extent this is due to the bees’ documented affinity for pollen from plants sharing their geographical origins [20,21,54] or simply a phenological match with common early- and late-season flowering ornamental plants in our region. All three species are polylectic for nectar [20,21,34] and we collected them from both non-native and some native plants. Most of the sampled woody plants that bloomed before 1 April, coinciding with the flight of *O*. *cornifrons* and *O*. *taurus*, or after 1 July, coinciding with flight of *M*. *sculpturalis*, were non-native. Such plants are popular for urban landscapes because they provide visual interest during periods when few native woody ornamentals are in bloom. Thus, the host associations of the non-native bees in our samples likely reflect, in part, the limited selection of native landscape plants that bloom early or late in the growing season when those bees are active.

## 5. Conclusions

Although flowering woody landscape plants provide many ecosystem services and are selected for a variety of reasons besides pollinator conservation [56], their value for supporting urban bees is increasingly recognized [8,9,57]. Many species and cultivars of non-native herbaceous and woody ornamental plants provide floral resources to both native and non–native bees [6,7,8,9,10,11,14,15,57,58,59]. Plants introduced into new regions tend to retain the phenology of their natal provenance, so incorporating some non-natives into urban landscapes can benefit bees by extending the flowering season. This study, which analyzed host associations of 11,275 bees sampled from 22 native and 23 non-native species of woody landscape plants, found six species of non-*Apis* non-native bees, collectively accounting for 3% of the total collections. Non-native non-*Apis* bees in total, and two invasive species, *Megachile sculpturalis* and *Osmia taurus*, in particular, were more abundant on non-native woody plants. However, most of the bees hosted by the non-native woody plants were either native species (73.7%) or *Apis mellifera* (21.5%). Whether the benefits of such plants to urban bee conservation outweigh the possibility that they might facilitate spread and adaptation of invasive bees to urban environments or otherwise disrupt urban pollination networks warrants further study.

## Figures and Tables

**Table 1:** Abundance of *Apis mellifera* (Am) and six other non-native bee species (Aw = *Andrena wilkella*, Hp = *Hylaeus punctatus*, Mr = *Megachile rotundata*, Ms = *Megachile sculpturalis*, Oc = *Osmia cornifrons*, and Ot = *Osmia taurus*) in samples from 45 species of bee-attractive trees and shrubs across 213 urban landscape sites (N = 5, 4, or 3 sample sites each for 35, 8, and 2 of the plant species, respectively).

					Non–Native Bees by Family ^c^						
					Apid	Andr	Coll	Megachilidae			
Species	Native or Not ^a^	Origin ^b^	Total Bees	Bloom Period	Am	Aw	Hp	Mr	Ms	Oc	Ot
*Cornus mas*	Nn	SEu/WAs	247	March	4	–	–	–	–	–	4
*Fothergilla gardenia*	Nn	NAm	267	March–April	3	–	–	–	–	–	–
*Malus* spp.	V	M	258	March–April	19	–	–	–	–	1	13
*Prunus* spp.	V	M	194	March–April	23	–	–	–	–	3	11
*Prunus subhirtella* ‘Pendula’	Nn	EA	285	March–April	53	–	–	–	–	18	32
*P. subhirtella* ‘Autumnalis’	Nn	EA	213	March–April	177	–	–	–	–	2	13
*Viburnum* × *burkwoodii*	Nn	EA	284	April	27	–	–	–	–	–	37
*Aesculus* × *carnea*	Nn	M	282	April–May	115	–	–	–	–	–	9
*Amelanchier* spp.	N	NAm	215	April–May	6	–	–	–	–	–	–
*Cercis canadensis*	N	NAm	274	April–May	0	–	–	–	–	3	4
*Cornus florida*	N	NAm	155	April–May	113	–	–	–	–	–	–
*Crataegus viridus*	N	NAm	345	April–May	52	–	–	–	–	–	–
*Ilex opaca*	N	NAm	242	April–May	51	–	–	1	–	–	–
*Ilex* × *attenuata*	N	NAm	302	April–May	139	–	–	2	–	–	–
*Ilex* × *meserveae*	Nn	M	254	April–May	100	–	–	–	–	1	12
*Nyssa sylvatica*	N	NAm	268	April–May	57	1	–	–	–	–	–
*Prunus laurocerasus*	Nn	EEu/WAs	273	April–May	2	–	–	–	–	–	1
*Prunus virginiana*	N	NAm	220	April–May	4	–	–	–	–	–	–
*Deutzia scabra*	Nn	EAs	245	May	36	–	–	2	–	–	–
*Pyracantha* spp.	Nn	Eu/WAs	238	May	3	–	–	–	–	–	–
*Amorpha fruticosa*	N	NAm	302	May–June	77	–	–	–	–	–	–
*Cladrastis kentukea*	N	NAm	268	May–June	182	5	–	–	–	–	–
*Philadelphu*s spp.	V	Eu/As/NAm	253	May–June	2	–	–	–	–	–	–
*Physocarpus opulifolius*	N	NAm	167	May–June	13	–	–	–	–	–	–
*Spirea virginiana*	N	NAm	277	May–June	2	3	–	–	–	–	–
*Syringa reticulata*	Nn	EAs	221	May–June	16	2	–	–	–	–	–
*Cephalanthus occidentalis*	N	NAm	199	June	7	–	–	–	–	–	–
*Ilex verticillata*	N	NAm	267	June	145	–	–	–	–	–	–
*Rosa setigera*	N	NAm	160	June	32	–	–	–	–	–	–
*Aesculus parviflora*	N	NAm	260	June–July	67	1	1	–	3	–	–
*Hypericum frondosum*	N	NAm	268	June–July	90	–	–	–	–	–	–
*Itea virginica*	N	NAm	270	June–July	53	2	–	2	–	–	–
*Koelreuteria paniculata*	Nn	EAs	282	June–July	120	1	1	1	4	–	–
*Oxydendrum arboreum*	N	NAm	228	June–July	10	–	–	6	9	–	–
*Tilia cordata*	Nn	Eu/WAs	264	June–July	127	–	–	–	–	–	–
*Maackia amurensis*	Nn	NAs/EAs	165	July	11	–	–	10	36	–	–
*Aralia elata*, *spinosa*	V	EAs/NAm	270	July–August	60	–	–	–	–	–	–
*Clethra alnifolia*	N	NAm	260	July–August	7	–	–	4	–	–	–
*Hydrangea paniculata*	Nn	EAs	283	July–August	71	–	–	–	–	–	–
*Lagerstroemia* sp.	Nn	EAs	220	July–August	58	–	–	–	–	–	–
*Rhus copallinum*	N	NAm	269	July–August	163	–	–	–	–	–	–
*Tetradium daniellii*	Nn	EAs	258	July–August	167	1	–	–	22	–	–
*Vitex agnus–castus*	Nn	SEu/WAs	263	July–August	6	–	–	–	23	–	–
*Abelia* × *grandiflora*	Nn	EAs	275	July–September	8	–	–	13	–	–	–
*Heptacodium miconioides*	Nn	EAs	265	August–September	34	–	–	1	–	–	–

^a^ N = native to North America; Nn = non-native to North America; V = varies. ^b^ NAm = North America; EEu, SEu = eastern or southern Europe; EAs, NAs, Was = eastern, northern, or western Asia. ^c^ Apid = Apidae, Andr = Andrenidae, Coll = Collectidae. Bee geographical origins (Russo 2016): *Apis mellifera*, *Andrena wilkella*, and *Hylaeus punctatus*: Europe; *Megachile rotundata*: Europe to China; *Megachile sculpturalis*: far east China, Korea, and Japan; *Osmia cornifrons* and *Osmia taurus*: east China and Japan.

**Table 2:** Summary of analysis of variance for effects of plant species, plant type (tree or shrub), provenance (native or non–native) on abundance of non–native bees.

	**All Non-Native Bees Other Than *Apis mellifera* ^1^**			***Apis mellifera***		
Source	df	*F*	Pr > *F*	df	*F*	Pr > *F*
Plant species	44	3.38	<0.001	44	5.82	<0.001
Plant family	20	3.39	<0.001	20	4.11	<0.001
Plant type	1	2.40	0.123	1	0.50	0.480
Provenance	1	17.41	<0.001	1	0.91	0.340
	***Megachile sculpturalis***			***Osmia taurus***		
Source	df	*F*	Pr > *F*	df	*F*	Pr > *F*
Plant species	44	5.99	<0.001	44	2.25	<0.001
Plant family	20	3.32	<0.001	20	3.15	<0.001
Plant type	1	0.12	0.726	1	6.04	0.015
Provenance	1	5.19	0.023	1	5.50	0.020

^1^ Includes *M. sculpturalis*, *O. taurus*, *Andrena wilkella*, *Hylaeus punctatus*, *Megachile rotundata*, and *Osmia cornifrons*). The latter four species were not separately analyzed due to small sample sizes.

## Data Availability

The dataset analyzed for this study is available at UKnowledge, the University of Kentucky’s open-access data repository: https://doi.org/10.13023/69rg-pn90 (accessed on 24 February 2022).

## Supplementary Materials

The following are available online at https://www.mdpi.com/article/10.3390/insects13030238/s1, Table S1: Families and full species names of the 45 flowering woody landscape plants from which bees were sampled.; Table S2: Number of non-native bees other than *Apis mellifera* in ca. 50-bee samples from flowering woody landscape plants, and number of sample sites at which those bees were found.
