# Supplementary material for: Non-Native Non-Apis Bees Are More Abundant on Non-Native Versus Native Flowering Woody Landscape Plants
Source: Insects. 2022 Feb 28;13(3):238. doi: 10.3390/insects13030238 (PMC8951211; doi:10.3390/insects13030238)
Supplement: Supplementary file 1 [file insects-13-00238-s001.zip › PotterMach.Insects.Table.S1.pdf]

Supplemental Table S1. Families and full species names of the 45 flowering woody landscape plants from which bees were sampled.

Family Adoxaceae

Viburnum × *burkwoodii* Burkwood & Skipw. (shrub)

Family Aquifoliaceae

Ilex × *attenuata* Ashe (shrub)

Ilex × *meserveae* SY Hu (tree)

Ilex opaca Aiton (tree)

Ilex verticillata (L.) A. Gray (shrub)

Family Araliaceae

Aralia elata, spinosa L. (tree)

Family Anacardiaceae

Rhus copallinum L. (tree)

Family Caprifoliaceae

Abelia × *grandiflora* (shrub)

Heptacodium miconioides Rehder. (tree)

Family Clethraceae

Clethra alnifolia L. (shrub)

Family Cornaceae

Cornus florida L. (tree)

Cornus mas L. (tree)

Family Ericaceae

Oxydendron arboretum (L.) (tree)

Family Fabaceae

Amorpha fructicosa L. (shrub)

Cercis canadensis L. (tree)

Cladrastis kentuckea Rudd (tree)

Maackia amurensis Rupr. & Maxim. (tree)

Family Hamamelidaceae

Fothergilla gardenia L. (shrub)

Family Hydrangeaceae

Deutzia scabra Thumb. (shrub)

Hydrangea paniculata Sieb. (shrub)

Philadelphus sp. L. (shrub)

Family Hypericaceae

Hypericum frondosum Michx. (shrub)

Family Iteaceae

Itea virginica L. (shrub)

Family Lamiaceae

Vitex agnus-castus L. (tree)

Family Lythraceae

Lagerstroemia sp. L. (tree)

Family Malvaceae

Tilia cordata Mill. (tree)

Family Nyssaceae

Nyssa sylvatica Marsh. (tree)

Family Oleaceae

Syringa reticulata (Blume) (tree)

Family Rosaceae

Amelanchier arborea (Michx. F.) (tree)

Crataegus viridis L. (tree)

Malus sp. L. (tree)

Physocarpus opulifolius (L.) Maxim. (shrub)

Prunus laurocerasus L. (shrub)

Prunus subhirtella 'Pendula' Miq. (tree)

Prunus subhirtella 'Autumnalis Pendula' Miq. (tree)

Prunus virginana L. (shrub)

Pyracantha sp. M. Roem. (shrub)

Rosa setigera Michx. (shrub)

Spiraea virginiana Britton (shrub)

Family Rubiaceae

Cephalanthus occidentalis L. (shrub)

Family Rutaceae

Tetradium daniellii (Benn.) T.G. Hartley (tree)

Family Sapindaceae

Aesculus × *carnea* Hayne (tree)

Aesculus parviflora Walter (tree)

Koelreuteria paniculata Laxm. (tree)
