# Supplementary material for: Non-Native Non-Apis Bees Are More Abundant on Non-Native Versus Native Flowering Woody Landscape Plants
Source: Insects. 2022 Feb 28;13(3):238. doi: 10.3390/insects13030238 (PMC8951211; doi:10.3390/insects13030238)
Supplement: Supplementary file 1 [file insects-13-00238-s001.zip › PotterMach.Insects.Table.S2.pdf]

Table S2. Number of non-native bees (N) other than *Apis mellifera* in ca. 50-bee samples from flowering woody landscape plants, and number of sample sites at which those bees were found. There were five sample sites for all plant species except *P. subhirtella* 'Autumnalis', *Prunus* spp., and *T. danielii* (four sites each), and *M. amurensis* (three sites).

Bee Species and Provenance[a]	Total	Plant Hosts	Plant Provenance	N	Sites found at[4]
Andrena wilkella	16	*Aesculus parviflora*	North America	1	1
Europe		*Cladastris kentuckea*	North America	5	3
		Itea virginica	North America	2	2
		Koelreuteria paniculata	East Asia	1	1
		Nyssa sylvatica	North America	1	1
		Spiraea virginiana	North America	3	2
		Syringa reticulata	East Asia	2	1
		Tetradium daniellii	East Asia	1	1
Hylaeus punctatus	2	*Aesculus parviflora*	North America	1	1
Europe		*Koelreuteria paniculata*	East Asia	1	1
Megachile rotundata	43	*Abelia × grandiflora*	East Asia	13	4
Europe		*Clethra alnifolia* '16 candles'	North America	4	2
		Deutzia scabra	East Asia	2	1
		Heptacodium miconioides	East Asia	1	1
		Ilex opaca	North America	1	1
		Ilex × attenuata	North America	2	2
		Itea virginica	North America	2	1
		Koelreuteria paniculata	East Asia	1	1
		Maackia amurensis	East Asia	10	1
		Oxydendrum arboreum	North America	6	1
		Vitex agnus-castus	Southern Europe	1	1
Megachile sculpturalis	97	*Aesculus parviflora*	North America	3	2
East Asia		*Koelreuteria paniculata*	East Asia	4	2
		Maackia amurensis	North/East Asia	36	3
		Oxydendrum arboreum	North America	9	3
		Tetradium daniellii	East Asia	22	3
		Vitex agnus-castus	Southern Europe	23	3
Osmia cornifrons	28	*Cercis canadensis*	North America	3	2
East Asia		*Ilex × meserveae*	Multiple origins[b]	1	1
		Malus spp.	Multiple origins[c]	1	1
		Prunus spp.	Multiple origins[d]	3	2

		Prunus subhirtella 'Pendula'	East Asia	2	1
		Viburnum burkwoodii	East Asia	18	3
Osmia taurus	136	*Aesculus* × *carnea*	Multiple origins[3]	9	3
East Asia		*Cercis canadensis*	North America	4	3
		Cornus mas	Southern Europe/ Western Asia	4	1
		Ilex × *meserveae*	Multiple origins[1]	12	3
		Malus spp.	Multiple origins[2]	13	5
		Prunus spp.	Multiple origins[2]	11	3
		Prunus laurocerasus	Eastern Europe/ Western Asia	1	1
		Prunus subhirtella 'Autumnalis'	East Asia	13	3
		Prunus subhirtella 'Pendula'	East Asia	32	4
		Viburnum burkwoodii	East Asia	37	4

[a]Provenance based on information in Russo (2016) and Droege (2021)

[b]Hybrid between *Ilex aquifolium* (Europe, North Africa, West Asia) and *I. rugosa* (East Asia)

[c]Includes multiple species with unknown or multiple origins

[d]Hybrid between *Aesculus hippocastanum* (SE Europe) and *A. pavia* (N America)
